# Temporal-Spatial Variation and Controls of Soil Respiration in Different Primary Succession Stages on Glacier Forehead in Gongga Mountain, China

**DOI:** 10.1371/journal.pone.0042354

**Published:** 2012-08-06

**Authors:** Ji Luo, Youchao Chen, Yanhong Wu, Peili Shi, Jia She, Peng Zhou

**Affiliations:** 1 Institute of Mountain Hazards and Environment, Chinese Academy of Sciences, Chengdu, China; 2 Graduate University of the Chinese Academy of Sciences, Chinese Academy of Sciences, Beijing, China; 3 Institute of Geographic Sciences and Natural Resources Research, Chinese Academy of Sciences, Beijing, China; Argonne National Laboratory, United States of America

## Abstract

Soil respiration (SR) is an important process in the global carbon cycle. It is difficult to estimate SR emission accurately because of its temporal and spatial variability. Primary forest succession on Glacier forehead provides the ideal environment for examining the temporal-spatial variation and controlling factors of SR. However, relevant studies on SR are relatively scarce, and variations, as well as controlling factors, remain uncertain in this kind of region. In this study, we used a static chamber system to measure SR in six sites which represent different stages of forest succession on forehead of a temperate glacier in Gongga Mountain, China. Our results showed that there was substantial temporal (coefficient of variation (CV) ranged from 39.3% to 73.9%) and spatial (CV ranged from 12.3% to 88.6%) variation in SR. Soil temperature (ST) at 5 cm depth was the major controlling factor of temporal variation in all six sites. Spatial variation in SR was mainly caused by differences in plant biomass and Total N among the six sites. Moreover, soil moisture (SM), microbial biomass carbon (MBC), soil organic carbon (SOC), pH and bulk density could influence SR by directly or indirectly affecting plant biomass and Total N. Q_10_ values (ranged from 2.1 to 4.7) increased along the forest succession, and the mean value (3.3) was larger than that of temperate ecosystems, which indicated a general tendency towards higher-Q_10_ in colder ecosystems than in warmer ecosystems. Our findings provided valuable information for understanding temporal-spatial variation and controlling factors of SR.

## Introduction

As the implications for the climate systems, carbon (C) cycle has received growing attention in recent year [1], [2]. Soils constitute the major C reserve in terrestrial ecosystems [3], and soil respiration (SR), which follows gross primary production (GPP) as the second largest C flux in the global terrestrial C cycle [4], is a critical ecosystem process that regulates C cycle in the earth system. Because of the large flux, even a small change in SR rate would have profound impact on the atmospheric CO_2_ concentration [5]. Therefore, accurately quantifying SR emission is crucial for the climate change projects. However, an obstacle to estimating SR emission accurately is its inherent temporal and spatial variability within and across ecosystems [6], [7], [8].

Soil temperature (ST) and soil moisture (SM) are reported as two main controlling factors on temporal variation of SR [9], [10]. Some biotic variables, such as microbial, root and litter biomass, may also strongly influence the seasonal variability of SR [11]. The spatial variation of SR has often been related to either the abiotic factors, such as ST, SM, soil organic carbon (SOC), soil total nitrogen (Total N), soil bulk density, soil porosity, soil pH, or biotic factors, such as plant root density, microbial biomass, litter amount [12], [13]. However, the accountability of these abiotic and biotic variables on temporal and spatial variation of SR will be dependent on the type of ecosystems being studied [14].

Forest succession is a fundamental ecological process which can modify biogeochemical cycles [15], ameliorate stand conditions and alter microclimate factors [16]. As forests develop from young to mature developmental stages, the differences in soil carbon pools, root biomass, soil microbial biomass, soil chemical and physical properties, etc, may cause SR varied within and across sites [17], [18], [19]. Many of the controlling factors compounded in SR change during the course of forest succession, thus more knowledge of SR changes along with a forest successional gradient is required. Primary forest succession can take place on glacier forehead, with receding ice cover presenting a chronosequence of development from the bare substrate to complex plant communities [20], [21]. As the forehead of a glacier is sensitive to climate change [22], forest succession on glacier forehead is the ideal environment for examining the SR and carbon cycle. However, few reports have been found on temporal-spatial variation and controlling factors of SR along forest succession on glacier forehead [23], especially for temperate glaciers.

**Figure 1:**
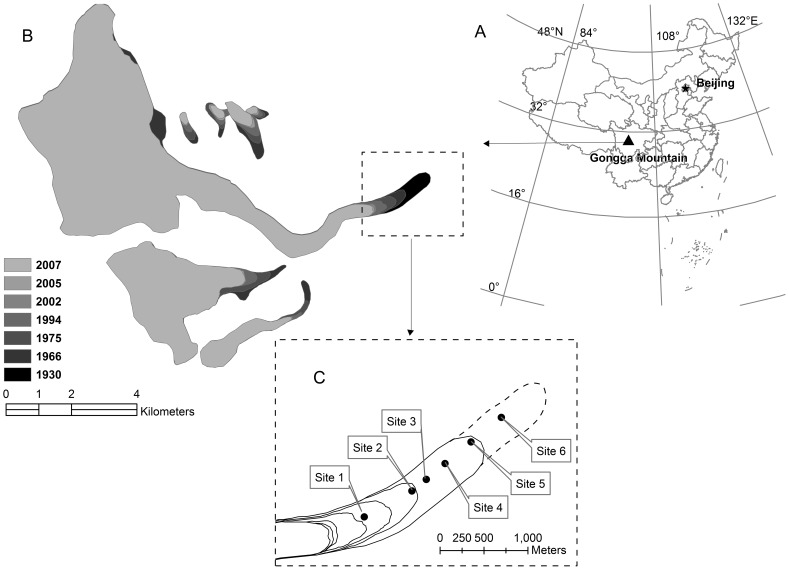
Location of study area. Location of Gongga Mountain (A), map of Hailougou Glacier (B) and approximate location of study sites (C). The dotted curve in panel C was adapted from Li et al. [24].

According to the Chinese Glacier Inventory, there are 8607 monsoonal temperate glaciers in China. Hailuogou Glacier, located on the east slope of Gongga Mountain in China, is one of temperate glaciers. Our study was conducted in stands representing different stages of primary succession on the forehead of Hailuogou Glacier. The objectives of this study were to (i) identify the temporal and spatial variation of SR in different stages of primary forest succession on the forehead of a temperate glacier; (ii) distinguish the controlling factors of temporal and spatial variation of SR within and across the succession stages.

**Table 1:** Some characteristics of the study sites in year 2009.

Site	1	2	3	4	5	6
Stand Age (year)	19	46	53	71	82	121
Plot size (m^2^)	100	100	100	100	100	100
Mean ST (°C)	6.2	6.1	5.8	5.4	5.1	5.4
Mean SM (%)	28.4	35	31.3	35.4	36.7	39.8
Biomass (kg·m^−2^)	5.93	22.25	26.05	34.69	36.21	39.25
Total N (g/kg)	1.39	1.38	1.56	1.4	2.58	2.69
SOC (g/kg)	23.93	28.67	28.31	29.81	59.63	60.56
MBC (mg/kg)	43.95	118.75	131.54	244.94	281.33	191.95
pH	6.4	5.4	5.6	5.2	4.8	4.3
Bulk Density (g·cm^−3^)	1.41	1.44	1.36	0.92	1.1	0.94
Litterfall (g C m^−2^ year^−1^)	44.14	75.26	53.34	57.12	56.28	63.91

ST means soil temperature at 5 cm depth; SM means soil moisture in the topsoil (0–20 cm); Biomass means plant biomass of stand which was extracted from unpublished data; Total N and SOC represent total nitrogen and soil organic carbon in the top 20 cm depth respectively; MBC means microbial biomass nitrogen in the top 20 cm depth soil.

## Materials and Methods

### Ethics Statement

All necessary permits were obtained for the described field studies. We carried out the study based on Alpine Ecosystem Observation and Experiment Station of Gongga Mountain, which belongs to the Institute of Mountain Hazards and Environment, Chinese Academy of Sciences & Ministry of Water Conservancy. We obtained the permissions from the station and institute to use the sample plots, our study had no harm to the environment and the field study did not involve endangered or protected species.

**Figure 2:**
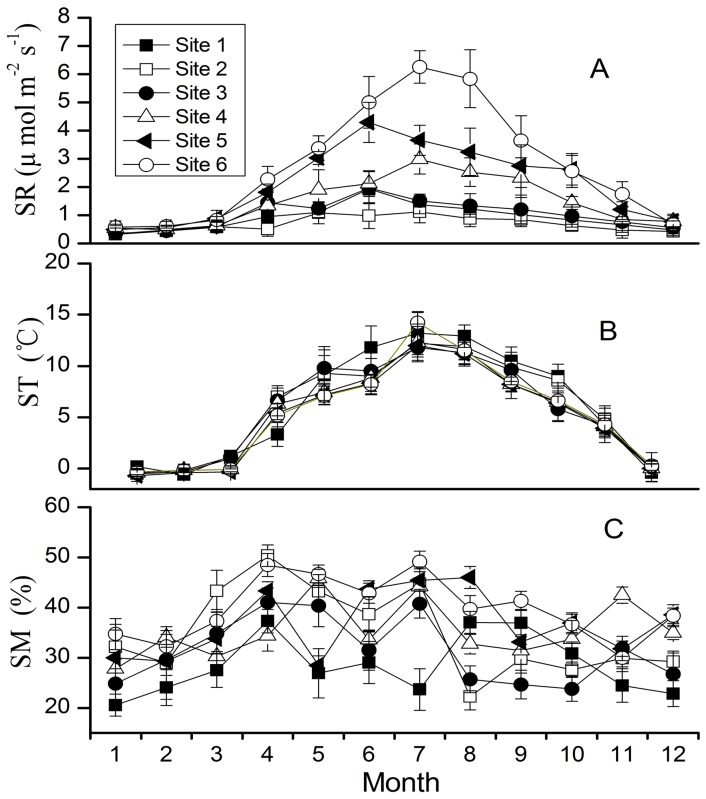
Temporal dynamics of SR (A), ST (B) and SM (C) for six sites. SR, ST and SM means soil respiration, soil temperature and soil moisture, respectively. The six sites represent different stages of forest succession on forehead of Hailougou Glacier in Gongga Mountain, China.

### Sites Description

Gongga Mountain (29°20′–30°20′N, 101°30′–102°15′E, 7556 m), located in the south-eastern fringe of Tibetan Plateau, is the highest peak in the eastern part of the Tibetan Plateau, and is one of the easternmost glacier areas in China (Fig. 1A). There are up to 74 glaciers distributed around Gongga Mountain, and Hailuogou Glacier, where our study was carried out, is the biggest one. Hailuogou Glacier is one of China’s monsoonal temperate glaciers, located on the east slope of Gongga Mountain, with the area of 25 km^2^. The regional climate is dominated by South Asian summer monsoon [24]. The mean annual air temperature is 3.8°C, the minimum mean air temperature is −4.3°C in January, and the maximum mean air temperature is 11.9°C in July. The total annual precipitation is about 2000 mm, most of which occurs from June to September.

**Figure 3:**
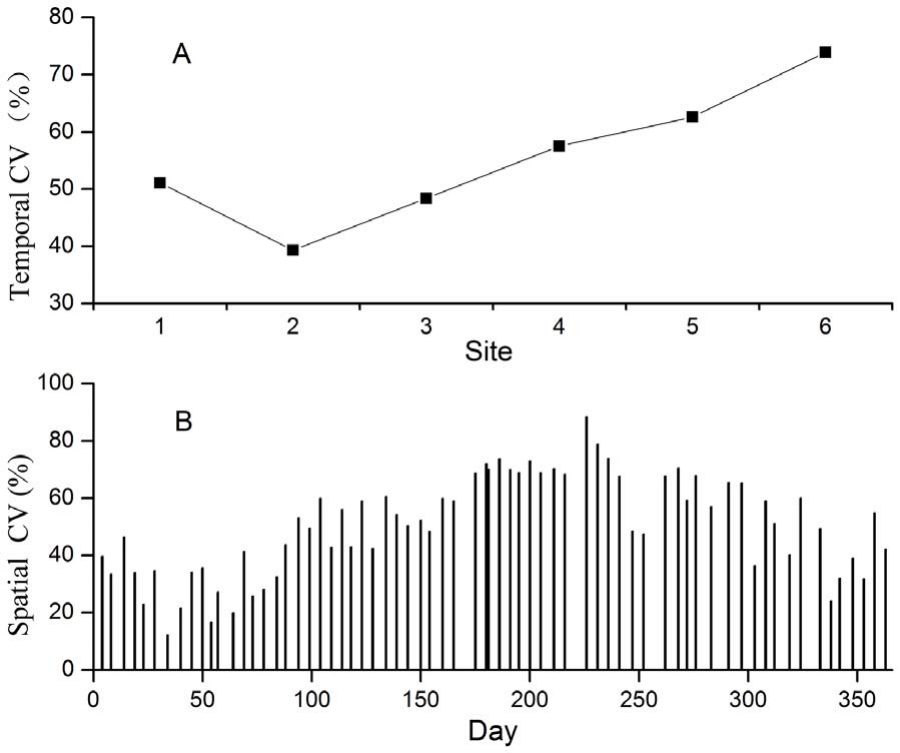
Temporal and spatial coefficient of variation (CV) of soil respiration. (A) Temporal CV of soil respiration for all six sites; (B) spatial CV of soil respiration during the whole year.

The observed recession of the Hailuogou Glacier began in about AD 1823 [25], and the recession has accelerated markedly since the early 20th century (Fig. 1B). Within about 2 km-long forehead of Hailuogou Glacier, a series of sites, representing different stages of vegetation succession, can be easily recognized. This study was conducted in six sites of this long-term primary succession (Fig. 1C). The six sites represent a sequence of successional stages from pioneer to climax vegetation communities [26]. The newly deglaciated moraine were well-drained, and gradually colonized by pioneer species including *Astragalus adsurgens, Salix* spp., *Hippophae rhamnoides,* and *Populus purdomii* (Site 1); *P. purdomii* grew quickly thus leading to species competition, and consequently a large number of *Salix* spp. and *H. rhamnoides* disappeared from the community (Site 2); *Betula utilis*, *Abies fabri* and *Picea brachytula* entered into the community (Site 3); The subdominant conifers increased in volume and eventually entered the canopy (Site 4); *P. purdomii* have been replaced by *A. fabri* and *P. brachytula* (Site 5); The forest has developed into a climax community dominated by *A. fabri* and *P. brachytula* (Site 6). Some characteristics of the stands are listed in Table 1.

**Figure 4:**
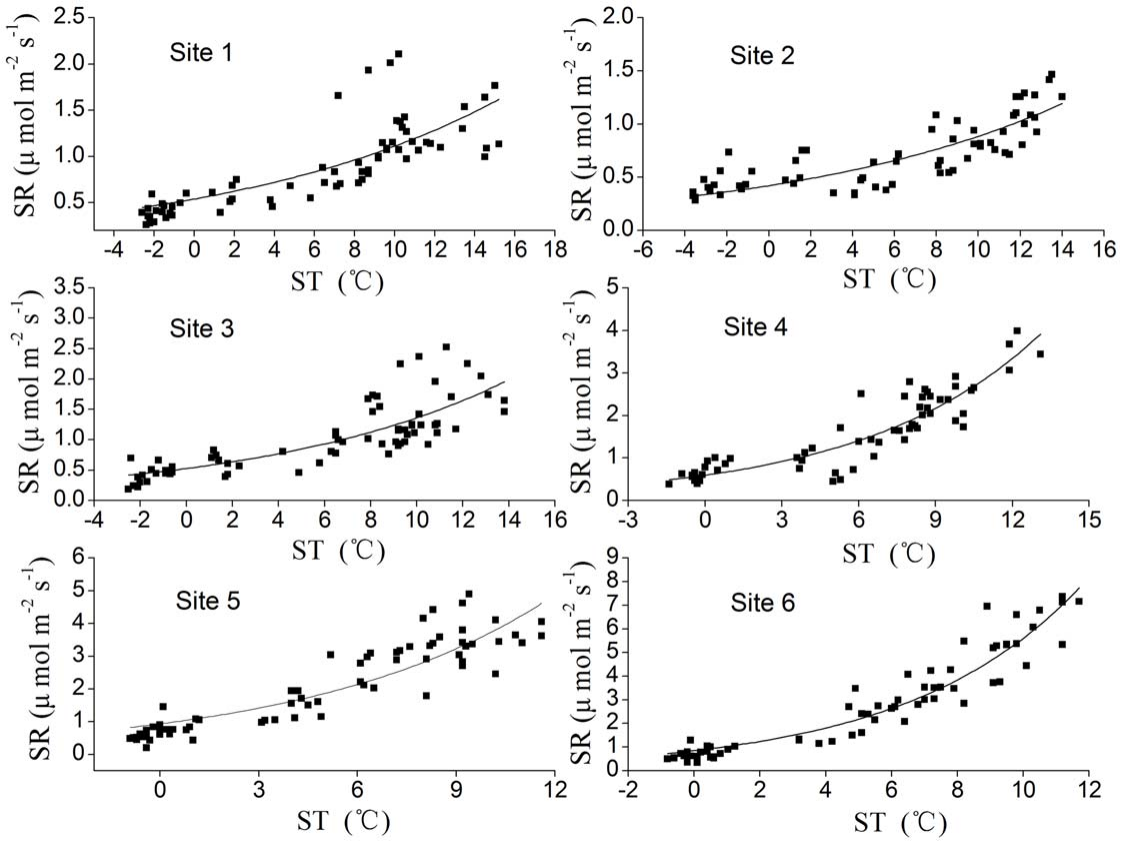
Relationship between SR and ST at 5 cm depth for six sites. SR and ST means soil respiration and soil temperature, respectively. The curves were fitted using equation 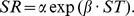

### SR, ST, SM and Litterfall Measurements

#### SR

Three 10 m×10 m plots were randomly located within each site. SR rates were measured about every 5 days in the year 2009, using a soil chamber (LI-6400–09, Li-Cor, Inc., Lincoln, NE) connected to a portable infrared gas analyzer (IRGA, Li-Cor, Inc., Lincoln, NE). To minimize soil surface disturbances, the chamber was mounted on polyvinyl chloride (PVC) soil collars inserted into the soil 2 or 3 cm. Three PVC collars were randomly placed in each plot one day before the measurements. A measurement consisted of placing the chamber on soil collar, scrubbing the CO_2_ to sub-ambient levels, and determining soil CO_2_ efflux over several 5-s periods. Data were recorded at 5-s intervals by the datalogger in the LI-Cor 6400 console. Each measurement usually took 1–3 min after placing the chamber on the collar. Concurrent measurements were made on the same day on each occasion at all six sites.

#### ST and SM

During the experimental period, soil temperature was monitored concurrently with SR using a copper/constantan thermocouple penetration probe (LI-6400–09 TC, LI-Cor) inserted into the soil to a depth of 5 cm in the vicinity of the SR chamber collars. The soil moisture content in the topsoil layer (0–20 cm) was measured gravimetrically and simultaneously with SR [27].

**Table 2:** Values of regression coefficient (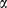 and β) and R^2^ in 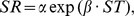 
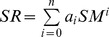
 and 
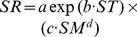
.

	ST				SM	ST-SM
Sites	_á_	â	R^2^	Q_10_	R^2^	R^2^
S1	0.539	0.083	0.585***	2.3	0.303***	0.595***
S2	0.420	0.075	0.713***	2.1	0.324***	0.732***
S3	0.525	0.095	0.641***	2.6	0.132***	0.672***
S4	0.593	0.144	0.845***	4.2	0.123***	0.847***
S5	0.930	0.138	0.798***	4.0	0.269***	0.804***
S6	0.845	0.156	0.903***	4.7	0.291***	0.910***

ST is soil temperature at 5 cm depth (°C), SM means soil moisture in the topsoil (0–20 cm), SR is monthly mean soil respiration (µ mol m^−2^ s^−1^), Q_10_ is calculated from equation Q_10_ = exp(10β), three asterisk means p<0.001.

#### Litterfall

Five 1 m×1 m litter traps were placed randomly in each plot and we collected litter from the traps monthly in 2009 for this study. All litter samples were cleaned, oven dried at 60°C for 48 h and recorded the final dry weight.

### Soil Sampling and Analysis

Three soil cores for chemical analyses, 20 cm in depth, were collected randomly from each plot in May and September 2009. The soil samples were divided into two parts: one was sieved to remove all visible plant material and air-dried for measurements of soil C and N concentration and pH value, the other was sieved and stored at 4°C until analysis for the estimation of microbial biomass carbon (MBC). SOC was determined using wet combustion as described by *Nelson and Sommers* [28], soil Total N was measured by semimicro-Kjedahl method [29], soil pH was measured with a potentiometer pH meter, MBC was determined by fumigation-extraction method [30]. Mean values were used for the later investigation.

**Figure 5:**
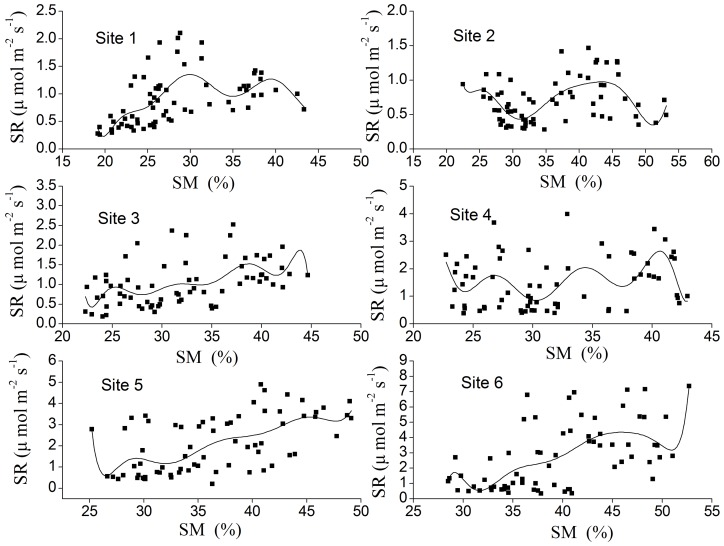
Relationship between SR and SM for six sites. SR and SM means soil respiration and soil moisture, respectively. The curves were fitted using equation 
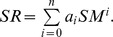

### Data Analysis

An exponential function was employed to describe the relationship between SR and ST [31]:
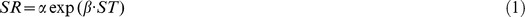
where 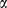 is the SR rate at 0°C (or a basal rate) and âis a parameter describing the temperature sensitivity (related to Q_10_ where 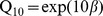).

Polynomial model was used to examine the relationship between SR and SM:
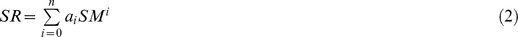
where 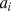 are constants, SM is soil moisture.

We developed an equation combining ST and SM to explain the SR. The best fits were obtained using an equation of the form:
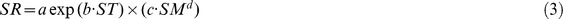



Coefficient of variation (CV) was used to quantify the temporal and spatial variability in SR:
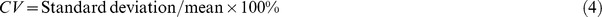



We applied monthly and yearly values of SR to further investigation. Monthly values were calculated as mean SR on the observation days each month. Then we used the monthly values to calculate the sum for the year. One-way ANOVA was used to compare differences in mean values of SR, ST and SM among the six sites. Pearson correlation analysis was used to examine the relationship between SR and ST/SM in each site. Liner regression model was applied to describe the relationship between mean annual SR and mean ST, mean SM, mean litterfall, biomass, SOC, Total N, MBC, pH and bulk density among the six sites. All statistical analyses were conducted using SAS 9.0.

**Figure 6:**
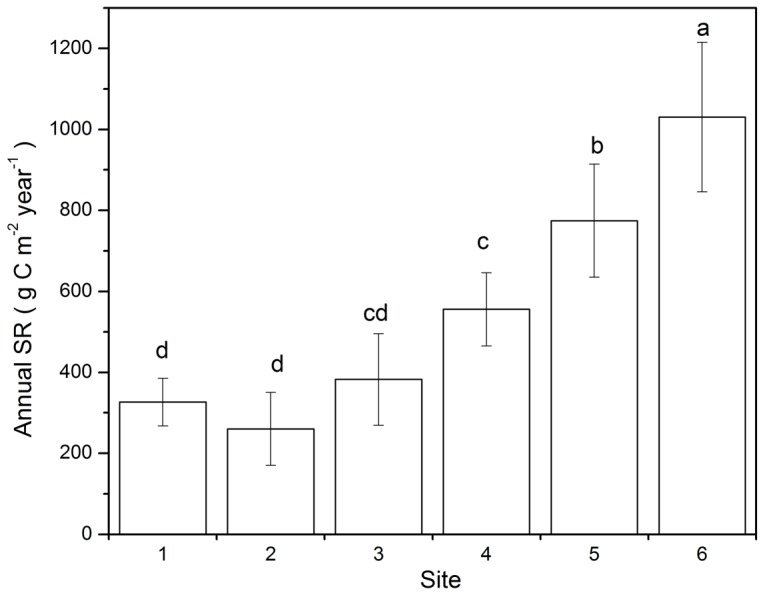
Annual SR for the six sites. SR means soil respiration. Different letters denote significant differences among means (á = 0.05) as determined by Turkey’s HSD test.

## Results

### Temporal Variation in Soil Respiration

As is shown in Fig. 2A, the patterns of temporal variation of SR were largely the same for the six sites. SR showed a similar single-peak pattern in all six sites in year 2009. SR was higher in the growing season than that in non-growing season. The lowest monthly SR rate was 0.32±0.05 µmol m^−2^ s^−1^ occurring at site 1 in January. By contrast, SR rate peaked in June or July in all the sites, with the maximum rate of 6.25±0.93 µmol m^−2^ s^−1^ in site 6.

The temporal coefficient of variation (CVs) of SR ranged from a minimum value of 39.3% for site 2 to a maximum value of 73.9% for site 6. The temporal CVs of SR appeared to be greater in the later successional stage than in the earlier stage (Fig. 3A).

**Figure 7:**
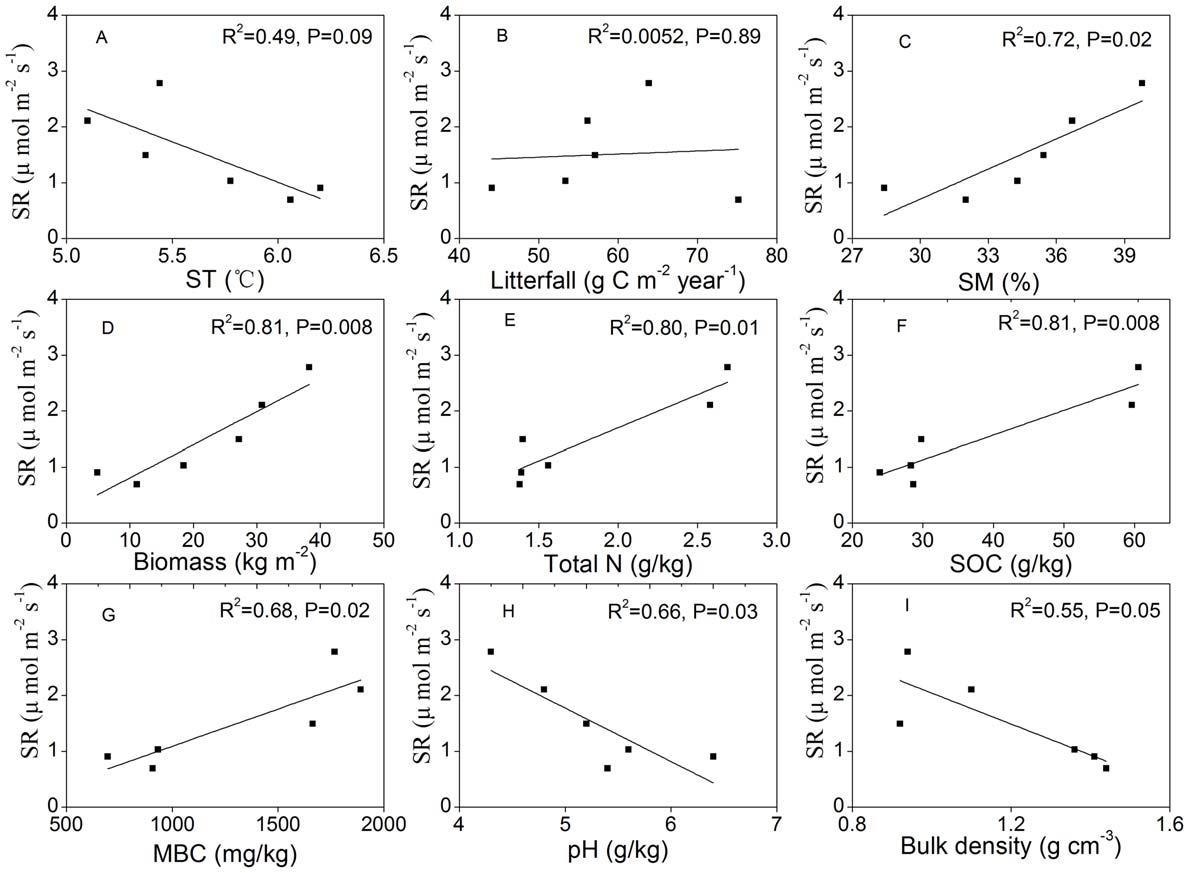
Relationships between mean SR and possible driving factors among sites. SR represented soil respiration; The driving factors were soil temperature (ST) at 5 cm depth (A), litterfall input (B), soil moisture (SM, C), plant biomass (D), total nitrogen (Total N, E), soil organic carbon (SOC, F), microbial biomass carbon (MBC, G), pH (H) and bulk density (I).

### Factors Controlling on Temporal Variation

SR experienced a distinct seasonal variation that paralleled the seasonality observed in ST at 5-cm depth (Fig. 2). SR increased exponentially with ST for all the sites over the whole year (Fig. 4). The ST-based regression models explained from 58.5% (site 1) to 90.3% (site 6) of the temporal variation in SR. The values of regression coefficient for all six sites were listed in Table 2.

Significantly positive correlation between SR and SM could be found in all the six sites (p<0.001). The polynomial functions were found to provide the best fit for the relationship between SR and SM in this study (Fig. 5). The SM-based models could explain the temporal variation in SR from 12.3% for site 4 to 32.4% for site 2. Values of R^2^ here are smaller than the corresponding R^2^ value using ST-based exponential model (equation (1)) for corresponding plots. When both SM and ST are used as independent variables and fitted to equation (3), we did not find a significant increase in R^2^ as compared with those fitted using equation (1) for each site (Table 2).

**Table 3:** Correlation coefficient of measured variables among the six sites.

	ST	Litterfall	SM	Biomass	Total N	SOC	MBC	pH
ST	1							
Litterfall	−0.053^∧^	1						
SM	−0.833*	0.336^∧^	1					
Biomass	−0.894*	0.215^∧^	0.980**	1				
Total N	−0.700^∧^	0.112^∧^	0.786^∧^	0.806*	1			
SOC	−0.755^∧^	0.203^∧^	0.817*	0.840*	0.988***	1		
MBC	−0.958**	0.172^∧^	0.867*	0.935**	0.755*	0.821*	1	
pH	0.783^∧^	−0.527^∧^	−0.959**	−0.934**	−0.803^∧^	−0.857*	−0.865*	1
Bulk density	0.829*	−0.050^∧^	−0.807*	−0.890**	−0.566^∧^	−0.621^∧^	−0.908*	0.751^∧^

ST, soil temperature; SM, soil moisture; Biomass, plant biomass; Total N, total nitrogen; SOC, soil organic carbon; MBC, microbial biomass nitrogen.

Caret, p>0.05;

one asterisk, p<0.05;

two asterisk, p<0.01;

three asterisk, p<0.001.

### Spatial Variation in Soil Respiration with Vegetation Succession

The mean SR rate tended to increase with the progress of succession stages for every month (Fig. 2A). The annual SR also tended to increase with the progress of primary succession: the later stages were significantly higher than the earlier stages (Fig. 6). The lowest annual SR rate was 260.1±90.2 g C m^−2^ year^−1^ in site 2, and the highest SR rate was 1030.8±184.6 g C m^−2^ year^−1^ in site 6.

The spatial CVs of SR among the six sites was higher in growing season than those in non-growing season (Fig. 3B). The highest mean spatial CV among the sites occurred at August, with a maximum value of 88.6%. While the lowest SR mean spatial CV was 12.3% occurring at February.

### Factors Controlling on Spatial Variation

Among the six sites, no significant correlation could be detected between mean annual SR rate and mean ST (R^2^ = 0.49, p = 0.09) (Fig. 7A), which could explain much of the temporal variation in SR for each site. However, SM, which was not a controlling factor of temporal variation in SR, significantly correlated with SR among the six sites (R^2^ = 0.72, p = 0.02) (Fig. 7C).

Simple regression analyses were carried out to investigate the relationship between other possible driving variables (soil properties, plant biomass, litterfall and MBC) and spatial variability of SR. The results showed that, among the six sites, mean SR rate positively correlated with plant biomass (R^2^ = 0.81, p = 0.008), Total N (R^2^ = 0.80, p = 0.01), SOC (R^2^ = 0.81, p = 0.008) and MBC (R^2^ = 0.68, p = 0.02) (Fig. 7D, 7E, 7F, 7G). In contrast, negative correlation can be detected between SR rate and pH (R^2^ = 0.66, p = 0.03), bulk density (R^2^ = 0.55, p = 0.05) (Fig. 7H, 7I). No significant correlation can be found between mean SR and mean yearly litterfall input (R^2^ = 0.0052, p = 0.89) (Fig. 7C).

## Discussion

### Temporal Variability of SR

In all the six sites, a main factor controlling on temporal variation of SR is ST. The exponential relationship between SR and ST (Fig. 4) was widely reported in previous studies [4], [32], [33], [34]. The ST-based model (equation (1)) can explain temporal variation on SR very well for each site (from 58.5% to 90.3%). The differences in ST-control along the forest succession stages may be caused by plant growth or soil microbial activities which may also contribute to temporal variations in SR [35], [36]. Q_10_ was used to describe ST dependence of SR. The Q_10_ values (Table 2), with a mean of 3.3, were higher than the median value of 2.4 in temperate ecosystems [4]. There seems to be a general tendency towards higher-Q_10_ in colder ecosystems than in warmer ecosystems, although the conclusion is a little up in the air for the exceptions in this study (Q_10_ in site 1 and site 2).

Apart from ST, SM is also recognized as the main factor controlling the temporal variability of SR [9], [14]. In this study, positive correlation between SR and SM can be detected in all the six sites. The relationship between SR and SM is complex, and both linear and nonlinear relationship have been reported [35], [37], [38]. In this study, polynomial equations best fitted the SR with SM. However, the resultant fits were low (Table 2), and the combined use of ST and SM functions did not improve our capability to better explain SR compared with the model based on ST only, thus indicated that ST exerts a stronger control than SM on SR in forests on forehead of Hailuogou Glacier. The effect of SM on temporal variability of SR was negligible in this region. The reason may be that, in the forehead of Hailuogou Glacier, the atmospheric humidity and SM content were high because of the frequent precipitation, and the temporal variation in SM for each site was small (CV from 14.9% in site 6 to 23.8% in site 2). SM availability is not the major controlling factor of temporal variation of SR in forests on forehead of Hailuogou Glacier.

### Spatial Variability of SR

Comparing to the factors controlling on temporal variation of SR, we found SM (Fig. 7C), rather than ST (Fig. 7A), was a major factor determining spatial variability of SR in different forest succession stages on forehead of Hailuogou Glacier. In the progress of forest succession, SM content tended to increase with the succession stages (Fig. 2C), for forest succession can affect the water-holding capacity of soil by increasing the SOC, improving soil construct, receding soil bulk density and enhancing soil porosity [39]. SM can affect SR through affecting water limitation of soil microbe and site plant productivity [40]. While the differences in ST among the six sites was not significant in this region (p>0.05), which made ST as the unimportant factor controlling on the spatial variation of SR.

We also found that plant biomass, Total N, SOC, MBC, pH and bulk density could contribute well to the spatial variation of SR among sites. Some study showed that SR rate negatively correlated with SOC and positively correlated with pH among the different stages of forest succession [41]. While in this study, we found that the annual mean SR was positively correlated with SOC (Fig. 7F), plant biomass (Fig. 7D), total N (Fig. 7E), and MBC (Fig. 7G), and negatively correlated with pH (Fig. 7H) and bulk density (Fig. 7I) among the six sites. As SR involves the process of converting organic into inorganic C, SR rate is untimely determined by the supply of C substrate [42]. The spatial dependence of SR on biomass has been reported in previous studies [4], [35]. As forest developed from pioneer to climax vegetation stand, plant biomass increased with this process. Plant biomass can affect SR by affecting soil C pool size derived from litterfall, root biomass and SOC [36], [40]. The correlation between SR and Total N content maybe explained by the dependence of plant growth and root activities on soil N availability. Soil pH can affect the SR through affecting activities of soil microbes. Microorganisms are generally considered as the driving force or catalyst behind the decomposition process [43], thus the magnitude of MBC may indicate potential rate of C flux [44]. The positive correlation between SR and MBC can also be found in previous studies [45], [46]. Bulk density tends to decrease with forest succession (Table 1). Lower soil bulk density contributes to higher soil porosity, thus resulting in higher soil oxygen availability which facilitates microbial activities [35], leading to the increase in SR along the succession stages. SR partly results from decomposition of ground litter [47], however, in this study, we did not find a significant correlation between annual SR and yearly litterfall input among sites (Fig. 7C). In early stages, the main components of litterfall was branches and leaves of deciduous tree, and in later stages, the main component was needles of spruce and fir, the difference in litterfall amount among sites was insignificant (p>0.05), thus caused the poor relationship between SR and litterfall.

As showed in Fig. 7, the single factor best explaining the spatial variation of SR among the different successional stages was plant biomass or soil properties such as Total N and SOC. Stepwise multiple regression analysis with all measured variables showed that plant biomass and Total N were the only variables in the model for SR (data not shown). The exclusion of the other factors may be accounted for the fact that most of site variables were highly autocorrelated (Table 3).

In this study, our estimates of Q_10_ had a tendency to increase with the process of succession (Table 2). Our finding is in contrast to that of Yi et al. [48] and Yan et al. [49], while is consistent with the research of Wang et al. [41]. The spatial variation of Q_10_ was found to correlate with ST, SM, SOC, MBC, and ecosystem types [50], [51]. In our study, SOC and MBC varied among successional stages, so the lower Q_10_ values in early stages might be ascribed to the lower SOC and MBC contents.

In summary, we investigated the temporal-spatial variation and the controlling factors of SR in different stages of forest succession on Hailuogou Glacier forehead in Gongga Mountain. Our results indicated that SR was high in growing season and low in non-growing season for all the six sites, and the total annual SR tended to increase with the progress of succession. ST was the main controlling factor of temporal variation of SR. In contrast, spatial variation in SR could be mainly attributed to plant biomass and Total N, while the other variables (SM, MBC SOC, pH and bulk density) could affect SR by directly or indirectly influencing plant biomass and Total N. Q_10_ values increased along the forest succession, and the mean value was larger than that of temperate ecosystems, which indicated a general tendency towards higher-Q_10_ in colder ecosystems than in warmer ecosystems. Our findings show that successional stage affects microclimate, plant and microbial biomass and soil properties, thus directly and indirectly influencing SR.
